# Comprehensive assessment of the acute lethal, risk level, and sub-lethal effects of four insecticides on *Trichogramma ostriniae*

**DOI:** 10.1371/journal.pone.0325733

**Published:** 2025-06-04

**Authors:** Wenya Zhu, Qiongqiong Guo, MengJiao Chen, Juan Wang, Ye Zhang, Ruiyan Ma

**Affiliations:** College of Plant Protection, Shanxi Agricultural University, Taiyuan, China; PMAS Arid Agriculture University: PMAS-Arid Agriculture University Rawalpindi, PAKISTAN

## Abstract

*Trichogramma ostriniae* is one of the most successfully used natural enemies in the integrated management of agroforestry pests. However, the extensive use of insecticides poses a significant threat to the survival and efficacy of *T. ostriniae*. To assess the compatibility of chemical pesticides with *T. ostriniae*, we investigated the acute toxicity, risk level, and sub-lethal effects of four insecticides (chlorfenapyr, emamectin benzoate, phoxim, and lambda-cyhalothrin) on reproduction, parasitism, detoxification enzymes, protective enzyme activities, and active substances under laboratory conditions. The results revealed that phoxim had the highest acute toxicity, with a median lethal concentration value of 2.8 × 10^−7^ mg/mL, whereas chlorfenapyr had the lowest at 5.06 × 10^−3^ mg/mL. Emamectin benzoate was classified as high risk, whereas the others were classified as extremely high risk. Insecticide exposure during the larval and pupal stages significantly reduced the emergence of *T. ostriniae* (*P* < 0.05). Lambda-cyhalothrin, emamectin benzoate, and chlorfenapyr extended the time required for prey-handling and reduced parasitism efficiency by 0.70%, 2.45%, and 4.50%. In contrast, phoxim increased the time required for prey-handling and improved parasitism efficiency by 25.37%. All insecticides affected protective enzyme activities, induced detoxification enzyme activity, reactive oxygen species, malondialdehyde and mitochondrial respiratory chain complex I levels, and decreased the adenosine triphosphate level. These findings underscore the differential impacts of insecticides on *T. ostriniae* and emphasize the need for cautious pesticide selection to balance pest control and natural enemy conservation, providing essential scientific guidance for sustainable agroforestry pest management.

## Introduction

*Trichogramma ostriniae* (Pang et Chen) (Hymenoptera: Trichogrammatidae) is one of the most successful natural enemies of egg parasitoids and has been bred and released in the field both domestically and internationally [1–3]. As an important natural enemy insect resource, *T. ostriniae* is primarily released in large quantities to achieve biological pest control [4], and has achieved significant success in managing agricultural and forestry pests [2,5]. The use of *T. ostriniae* in combating Asian corn borers*, Ostrinia furnacalis* (Guenée) (Lepidoptera: Crambidae), has emerged as a key component of integrated pest management (IPM) strategies for corn pests in China [6]. Owing to its numerous advantages as a biocontrol agent, *T. ostriniae* has attracted increasing attention.

Lambda-cyhalothrin, chlorfenapyr, emamectin benzoate, and phoxim are all used to control Lepidoptera pests [7–9], which is consistent with the goal of *Trichogramma* in controlling pests such as *O. furnacalis*. In IPM, when the pest population is large and it is difficult to effectively control pest damage quickly using biological control methods alone, such as *Trichogramma*, these insecticides can be used as emergency control measures to rapidly reduce the pest population and alleviate damage to crops [10]. Nevertheless, overuse of insecticides can potentially have detrimental effects on both the environment and human health [11,12], and also adversely affect non-target beneficial arthropods, including *Trichogramma* parasitoids [13,14]. Pesticides can have both lethal and sub-lethal effects on natural enemies, leading to mortality and adverse effects on various biological parameters, including developmental duration, adult longevity, morphology, predation ability, reproductive capacity (encompassing oviposition and fertility), movement, information exchange, and parasitic rate [15–17]. Therefore, the adverse effects of pesticides on natural enemies need to be investigated to refine chemical and biological control strategies and ultimately minimize the negative consequences on non-target species [18].

A comprehensive assessment of the lethal and sub-lethal effects of insecticides is imperative to thoroughly evaluate their risks to non-target organisms. We hypothesize that the acute and sub-lethal toxicities of the four insecticides (lambda-cyhalothrin, chlorfenapyr, emamectin benzoate, and phoxim) and their effects on *T. ostriniae* are different. Therefore, we investigated the acute and sub-lethal effects of these insecticides on reproduction, parasitism, and parasitism efficiency. Additionally, the activities of detoxification and protective enzymes were measured, along with the levels of malondialdehyde (MDA), reactive oxygen species (ROS), mitochondrial respiratory chain complex I (MRCC I), and adenosine triphosphate (ATP). This study provides crucial information for understanding the mechanisms associated with the sub-lethal effects of insecticides on *T. ostriniae*, which may aid in the incorporation of insecticides into IPM systems including *T. ostriniae*.

## Materials and methods

### Insects

*T. ostriniae* was collected from the parasitized eggs of *O. furnacalis* in maize fields located in Xinzhou City, Shanxi Province, China (112°44’2.400“ E, 38°25’0.120” N). These maize fields are experimental fields owned and managed by our institution. Therefore, no external permits were required for the collection of samples. After hatching, the *T. ostriniae* population was maintained under controlled laboratory conditions at temperatures of 24–26 °C, a relative humidity of 70–80%, and photoperiod of 15 L: 9 D. *Corcyra cephalonica* (Stainton) eggs were used as hosts for the breeding of *T. ostriniae*. The *C. cephalonica* population was reared in the laboratory for several generations, without pesticide exposure. Subsequently, fresh and clean *C*. *cephalonica* eggs were collected and subjected to ultraviolet irradiation for 30 min to terminate the embryos. Thereafter, these UV irradiated eggs were placed on 4.0 cm × 2.5 cm card paper that had been treated with white latex to ensure that the eggs did not clump together or overlap. Non-parasitic egg cards were obtained after drying.

### Insecticides and reagents

Emamectin benzoate (96%) and chlorfenapyr (95.7%) were obtained from Qingdao Runsheng Agrochemicals Technology Co., Ltd. (Qingdao, China). Phoxim (90.4%) was obtained from Shandong Bainong Sida Biotechnology Co., Ltd. (Shandong, China), and lambda-cyhalothrin (96%) was acquired from Yangzhou Suling Agrochemicals Technology Co., Ltd. (Yangzhou, China).

Enzyme-linked immunosorbent assay (ELISA) kits for carboxylesterases (CarEs), cytochrome P450 monooxygenases (CYPs), glutathione S-transferases (GSTs), catalase (CAT), superoxide dismutase (SOD), peroxidase (POD), ROS, MDA, MRCC I, and ATP were purchased from Shanghai Meilian Biotechnology Co., Ltd. (Shanghai, China).

### Bioassay

The residual film in the glass tube method was used to determine the acute toxicity of the four insecticides to the *T. ostriniae* population. The concentration range of the insecticides (emamectin benzoate: 1 × 10^−4^ mg/mL to 5 × 10^−3^ mg/mL, chlorfenapyr: 3 × 10^−4^ mg/mL to 5 × 10^−3^ mg/mL, phoxim: 6 × 10^−8^ mg/mL to 2 × 10^−6^ mg/mL, lambda-cyhalothrin: 7.5 × 10^−6^ mg/mL to 2 × 10^−4^ mg/mL) were determined by conducting preliminary tests. Each insecticide was serially diluted to five different concentrations using acetone. The test solution (0.5 mL) for each insecticide concentration was then added to a glass test tube (1.5 × 6 cm). The test tubes were subsequently placed in an unheated hot-dog machine (Guangzhou Tiancheng Food Machinery Co., Ltd., Guangzhou, China) that was operated at a constant speed until acetone was completely evaporated. This process ensured that the insecticides were evenly coated on the inner walls of the glass test tubes. The control group was treated with acetone. At 6 h after emergence, *T. ostriniae* adults were transferred into acetone-volatilized test tubes, and a small amount of 10% honey solution was added as a food source for emerging adults. This was done by gently drawing a thin line of the honey solution on the inner wall of the test tube using a sterile dissecting needle. The opening of each treated tube was sealed with a black cloth to permit air exchange, and the tubes were maintained at a temperature of 24–26 °C, relative humidity of 70–80%, and photoperiod of 15 L: 9 D. One hundred *T. ostriniae* adults were exposed to each concentration, with four replicates per treatment. After 8 h, the number of surviving and dead adults was recorded, and the median lethal concentration (LC_50_) was calculated.

### Safety assessment

The LR_50_ of the insecticides against *T. ostriniae* was assessed under controlled indoor conditions. The safety factor was derived from the ‘National Standards of the People’s Republic of China, Test guidelines on environmental safety assessment for chemical pesticides, Part 17: *Trichogramma* acute toxicity test (GB/T 31270.17-2014)’. The recommended field application dose of pesticides in China provided by the China Pesticide Information Network was used to calculate the safety factor, using the following formula:

Safety factor = LR_50_ (g a.i./ha)/ recommended field application concentration (g a.i./ha),

The risks posed by pesticides on natural enemies were assessed using a safety factor. Consequently, the risk levels of pesticides in *T. ostriniae* were categorized into four levels: (1) low risk with a safety factor > 5, (2) medium risk with a safety factor between 5 and 0.5, (3) high risk with a safety factor between 0.5 and 0.05, and (4) extremely high risk with a safety factor of ≤ 0.05.

### Sub-lethal effects of four insecticides on *T. ostriniae* at different developmental stages

*T. ostriniae* adults were removed after 4 h of exposure to an insect-egg ratio of 1:20 and then placed under conditions of temperature of 25 ± 1 °C, relative humidity of 75 ± 5%, and a photoperiod of 15 L: 9 D. The developmental stages of *T. ostriniae* were determined by dissecting parasitized *C. cephalonica* eggs for microscopic examination. Parasitized *C. cephalonica* eggs at different developmental stages, such as egg (8 h after incubation), larval (48 h after incubation), pre-pupal (92 h after incubation), and pupal (144 h after incubation) stages, were immersed in the test solution for 5 seconds, then removed, dried, and placed in glass tubes sealed with a black cloth. After emergence, the number of eggs that had been parasitized and the number of eggs from which adults had successfully emerged were counted under a dissecting microscope. Thereafter, the emergence rate was calculated. The acetone treatment was used as the control, and each treatment was replicated four times.

### Effects of four insecticides on parasitism, functional responses, and searching efficiency

The functional response of *T. ostriniae* to *C. cephalonica* eggs was evaluated in glass tubes by exposing *T. ostriniae* to a 50% lethal concentration (LC_50_) of the insecticide. The protocol for treating the parasitoid wasp is the same as described in the bioassay. After 8 h, female *T. ostriniae* adults that survived the treatment with the insecticide were transferred to new glass tubes which were pre-placed with eight *C. cephalonica* egg densities (20, 30, 50, 60, 100, 120, 150, and 200) on the cards with one *T. Ostriniae* in each tube. After 24 h, *T. ostriniae* was removed, and the tubes were sealed with black cloth, maintaining the same conditions until the end of the development process. Each treatment was repeated 20 times. The number of parasitized eggs was recorded after 5 days, with the blackening of *C. cephalonica* eggs being regarded as the criterion for parasitism.

The relationship between the parasitism rate and host density, based on the number of parasitized *C. cephalonica* eggs, was modeled using the least-squares method according to the Holling II model, resulting in the parasitism functional response equation. The functional response disk equation is expressed as N_a_ = aTN/ (1 + aT_h_N), where N_a_ represents the number of parasitized hosts, a is the instantaneous attack rate, N is the initial host density, T is the total available time, and T_h_ is the prey-handling time (24 h). The searching efficiency (S) was calculated using the formula S = a’/ (1 + a’T_h_N), where S denotes the searching efficiency, a’ is the rate of successful attacks, T_h_ is the prey-handling time, and N is the prey density [19]. The theoretical maximum parasitism rate is given by N_amax_ = 1/ T_h_. A smaller a’/ T_h_ indicates a weaker ability of natural enemies to control prey.

### Effects of four insecticides on protective enzyme activity and active substance content

Adult *T. ostriniae* specimens that survived 8 h after treatment with the LC_50_ concentrations of insecticides were selected for protective enzyme activity and active substance content analyses. Surviving *T. ostriniae* adults were frozen and pulverized using liquid nitrogen. A 10% homogenate was created by adding a nine-fold volume of saline solution. The supernatant was collected by centrifugation at 2000 rpm for 15 min at 4 °C. The levels of POD, SOD, CAT, GST, CarE, CYP, ROS, MDA, ATP, and MRCC I were measured using ELISA kits (AndyGene). This procedure was conducted in triplicate.

### Statistical analysis

All data were analyzed using SPSS software (version 16.0; SPSS Inc., Chicago, IL, USA) and plotted using the Origin software. The normality of all data was first checked before analysis and data that followed a normal distribution were presented as mean ± standard error (SE, n = 3). The homogeneity of variances was evaluated through the application of Levene’s test. One way analysis of variance was performed with Tukey test (homogeneity of variance) and Dunnett T3 (nonheterogeneity of variance) post hoc tests to examine the differences on acute toxicity, enzyme activities MDA, ROS, MRCC I and ATP levels. Statistical significance was set at *P* < 0.05.

## Results

### Acute toxicity and safety factors of four insecticides in T. ostriniae population

The results revealed that phoxim demonstrated the highest acute toxicity toward *T. ostriniae*, with an LC_50_ value of 2.8 × 10^−7^ mg/mL. Conversely, chlorfenapyr exhibited the lowest acute toxicity, with an LC_50_ value of 5.06 × 10^−3^ mg/mL (Table 1). The safety factor for emamectin benzoate was 9 × 10^−2^, while those for chlorfenapyr, phoxim, and lambda-cyhalothrin were 1.87 × 10^−2^, 3.7 × 10^−7^, and 6 × 10^−3^, respectively (Table 2). According to GB/T 31270.17–2014, emamectin benzoate is classified as a high-risk insecticide, whereas chlorfenapyr, lambda-cyhalothrin, and phoxim are classified as extremely high-risk insecticides (Table 2).

**Table 1:** Acute toxicity of insecticides in adult *T. ostriniae.*

Insecticides	LC_50_/(mg/ml)	Toxicity regression equation	Confidence interval of 95%	*χ* ^*2*^	*P*
**Emamectin benzoate**	2.44 × 10^−4^	Y = 3.757 + 1.045X	1.18 × 10^−3^ ~ 3.52 × 10^−4^	2.16	0.97
**Chlorfenapyr**	5.06 × 10^−3^	Y = 0.419 + 0.183X	0.07 × 10^-3^-2.9 × 10^−2^	1.21	0.84
**Phoxim**	2.80 × 10^−7^	Y = 11.40 + 1.742X	1.58 × 10^−7^ ~ 9.67 × 10^−8^	5.87	0.71
**Lambda cyhalothrin**	1.80 × 10^−5^	Y = 4.640 + 0.977X	1 × 10^−5^ ~ 2.7 × 10^−5^	4.67	0.90

**Table 2:** Safety evaluation of insecticides in adult *T. ostriniae.*

Insecticides	Safety factor	Safety grade
**Emamectin benzoate**	9 × 10^−2^	High risk
**Chlorfenapyr**	1.87 × 10^−2^	Extremely high risk
**Phoxim**	3.7 × 10^−7^	Extremely high risk
**Lambda cyhalothrin**	6 × 10^−3^	Extremely high risk

### Toxicity of four insecticides against *T. ostriniae* at different developmental stages

**Fig 1:**
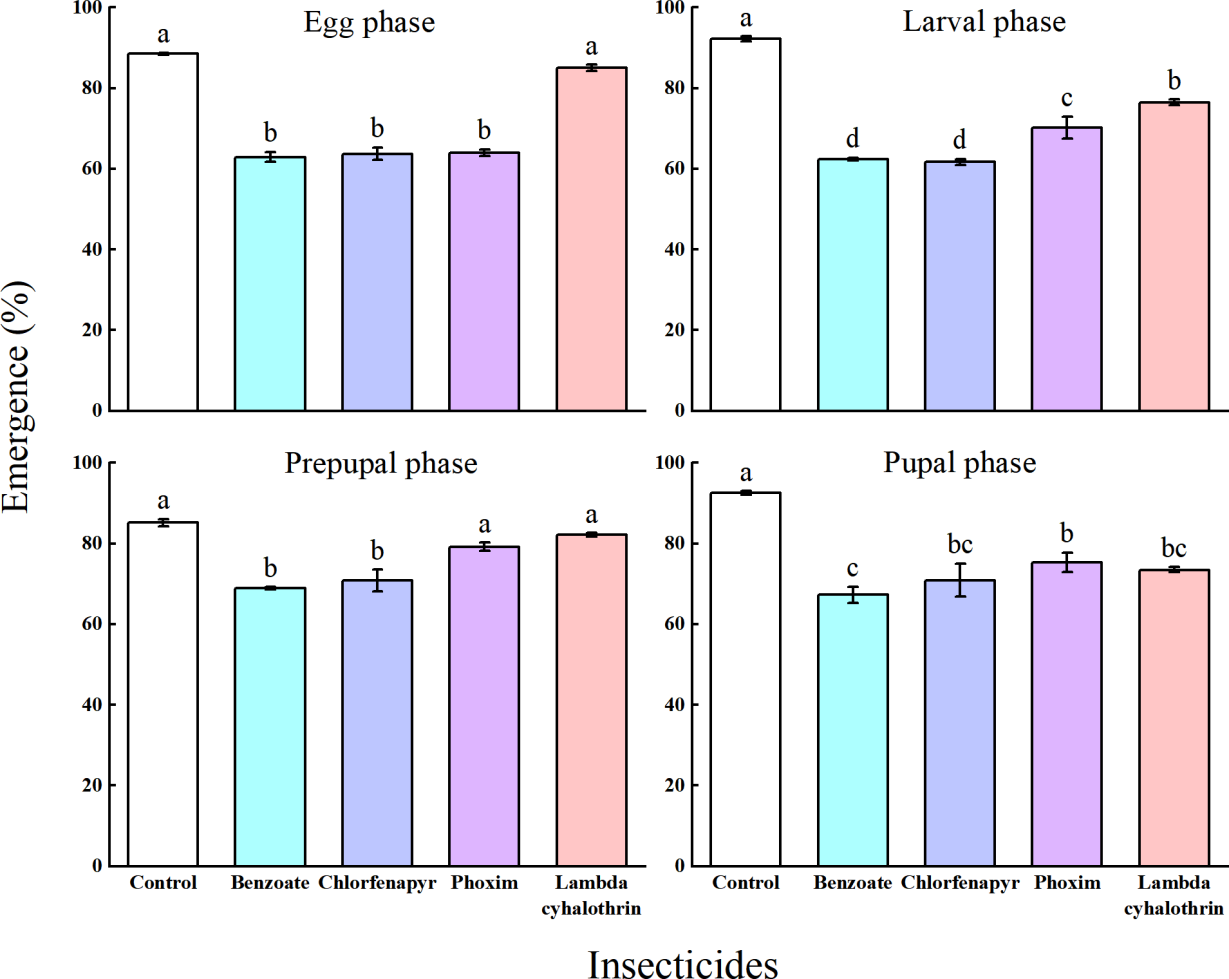
Emergence rates of *T. ostriniae* population exposed to insecticides at the egg (A), larval (B), pre-pupal (C), and pupal (D) stages. Data are expressed as mean ± standard error. Groups denoted by different letters represent significant differences (*P* < 0.05), whereas those denoted by the same letters indicate non – significant differences.

During the egg stage, the emergence rate of *T. ostriniae* post-treatment with chlorfenapyr, emamectin benzoate, and phoxim was significantly decreased compared with the control (*P* < 0.05), while Lambda cyhalothrin did not significantly affect the emergence rate (*P* > 0.05) (Fig 1A). A significant decrease in the emergence rate was observed in all four insecticides relative to the control group when they were applied at the larval stage (*P *< 0.05, Fig 1B). When applied at the pre-pupal stage, the emergence rate of *T. ostriniae* was significantly reduced by emamectin benzoate and chlorfenapyr, but not by phoxim and lambda-cyhalothrin (Fig 1C). Moreover, all four insecticides significantly decreased the emergence rate when were applied at the pupal stage (*P* < 0.05, Fig 1D).

### Effects of four insecticides on functional response and searching efficiency of *T. ostriniae*

The Holling II equation was used to model the parasitism rate of *T. ostriniae* at various *C. cephalonica* egg densities. Compared to the control group, the prey-handling time for *C. cephalonica* eggs by *T. ostriniae* was extended by lambda-cyhalothrin, emamectin benzoate, and chlorfenapyr, whereas it was shortened by phoxim. Relative to the control group, the parasitism efficiency of *T. ostriniae* decreased by 0.70%, 2.45%, and 4.50%, and the maximum parasitism number decreased by 10%, 18.65%, and 22.44%, when treated with lambda-cyhalothrin, emamectin benzoate, and chlorfenapyr, respectively. Conversely, phoxim treatment led to a 25.37% increase in parasitism efficiency and an 8.81% increase in maximum parasitism number (Table 3).

**Table 3:** Effects of insecticides on functional response of *T. ostriniae.*

Insecticides	Disc equation of functional response (*N*_a_=)	*R* ^²^	Instantaneous attack rate (*ɑ*)	Prey handling time (*T*_h_)	Parasitic efficiency (*ɑ*/*T*_h_)	Maximum parasitizing capacity (*N*_a_ max)
**Control**	1.0569*N*/(1 + 0.0185*N*)	0.843	1.0569	0.01748	60.46	57.21
**Emamectin benzoate**	1.2652*N*/(1 + 0.0275*N*)	0.768	1.2675	0.02724	58.98	46.54
**Chlorfenapyr**	1.3014*N*/(1 + 0.0293*N*)	0.874	1.3014	0.02254	57.74	44.37
**Phoxim**	1.1796*N*/(1 + 0.0175*N*)	0.903	1.2175	0.01606	75.80	62.25
**Lambda cyhalothrin**	1.1702*N*/(1 + 0.0228*N*)	0.865	1.1702	0.01949	60.03	51.30

**Fig 2:**
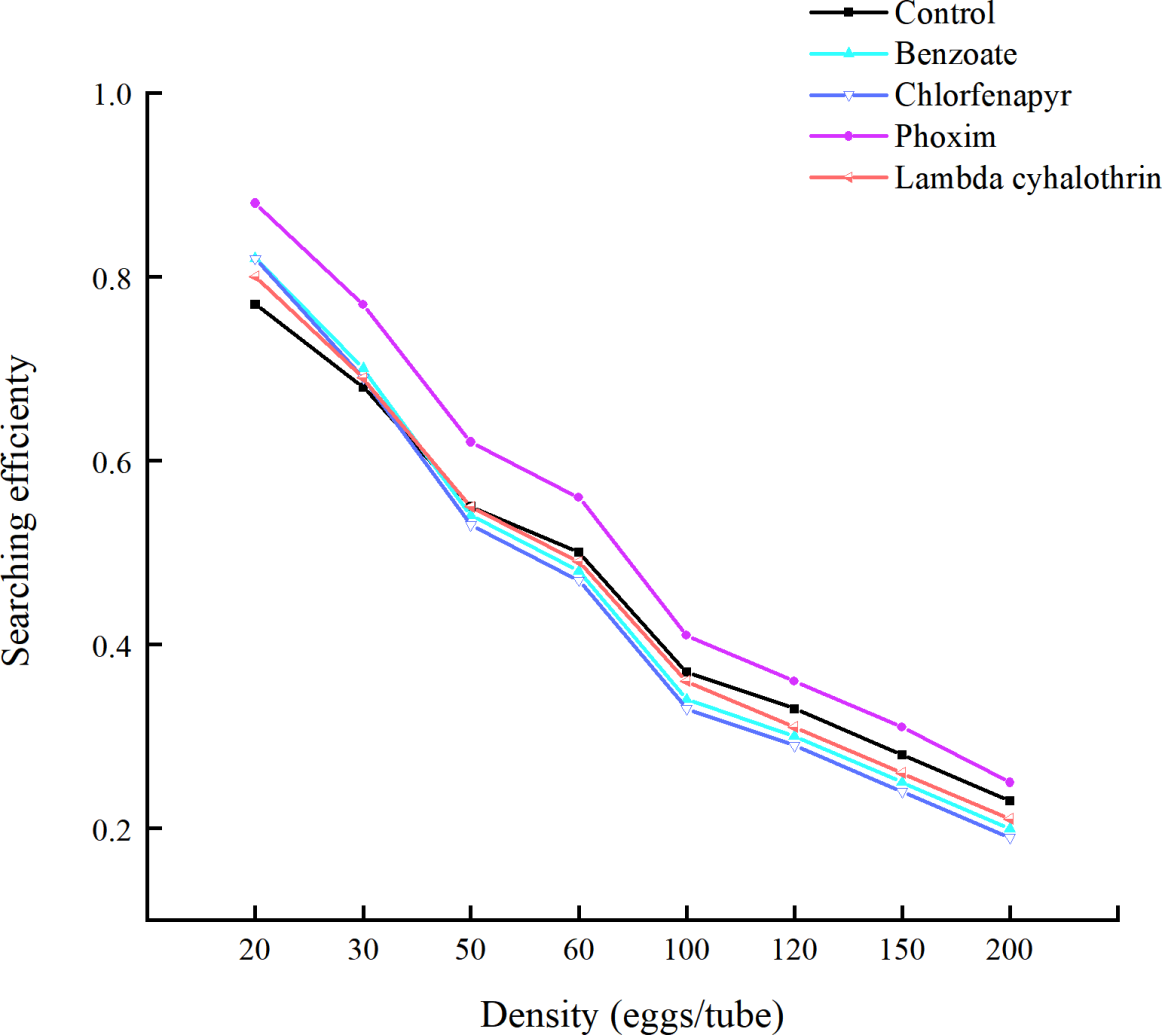
Sublethal effects of the four insecticides on the searching efficiency of *T. ostriniae.*

Searching efficiency, a critical behavioral trait of natural enemies, was found to be inversely related to the density of *C. cephalonica* eggs. As egg density increased, the search efficiency of *T. ostriniae* decreased. Among the four insecticides tested, phoxim had the most pronounced effect on the searching efficiency (Fig 2).

### Effects of four insecticides on protective enzyme activity of *T. ostriniae*

**Fig 3:**
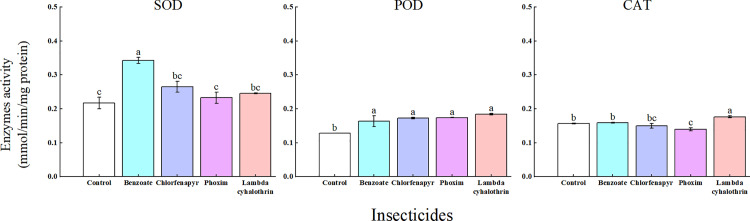
Sub-lethal concentration effects of the four insecticides on SOD, POD, and CAT activity in adult *T. ostriniae.* Data are expressed as mean ± standard error, and multiple comparisons among groups were performed using Tukey test. Groups denoted by different letters represent significant differences (*P* < 0.05), whereas those denoted by the same letters indicate non – significant differences (*P* > 0.05). SOD, superoxide dismutase; POD, peroxidase; CAT, catalase.

Significantly elevated SOD activity was observed in the treatment group compared to the control group following emamectin benzoate treatment (*P* < 0.05; Fig 3). All four insecticides induced POD activity in the following order: lambda-cyhalothrin, phoxim, chlorfenapyr, and emamectin benzoate. Phoxim significantly inhibited CAT activity in *T. ostriniae*, whereas lambda-cyhalothrin significantly induced CAT activity compared with that in the control group (*P *< 0.05; Fig 3). However, emamectin benzoate and chlorfenapyr had no significant effect on CAT activity (*P *> 0.05; Fig 3).

### Effects of four insecticides on detoxification enzyme activities of *T. ostriniae*

**Fig 4:**
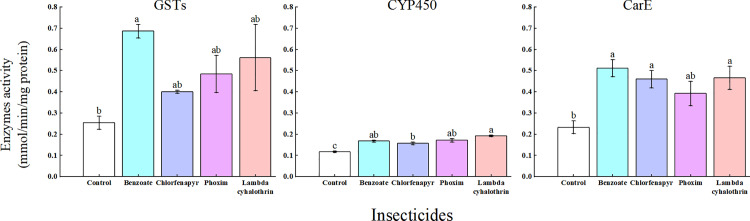
Sub-lethal concentration effects of the four insecticides on GST, CYP, and CarE activity in *T. ostriniae* population. Data are expressed as mean ± standard error and multiple comparisons among groups were performed using the Tukey test. Groups denoted by different letters represent significant differences (*P* < 0.05), whereas those denoted by the same letters indicate non – significant differences (*P* > 0.05). GST, glutathione-S-transferase; CYP, cytochrome P450; CarE, carboxylesterase.

Relative to the control group, the activities of CarEs, GSTs, and CYP450 in *T. ostriniae* were significantly increased following treatment with phoxim, chlorfenapyr, and emamectin benzoate (*P *< 0.05; Fig 4). Similarly, the activities of CarE and CYP in *T. ostriniae* significantly increased after treatment with lambda-cyhalothrin relative to those in the control group (*P *< 0.05; Fig 4). Notably, GST activity in the emamectin benzoate treatment group was the highest among all groups. Furthermore, GST activity in the emamectin benzoate group increased 2.71-fold relative to that in the control group (Fig 4).

### Effects of four insecticides on the MDA and ROS levels in *T. ostriniae*

**Fig 5:**
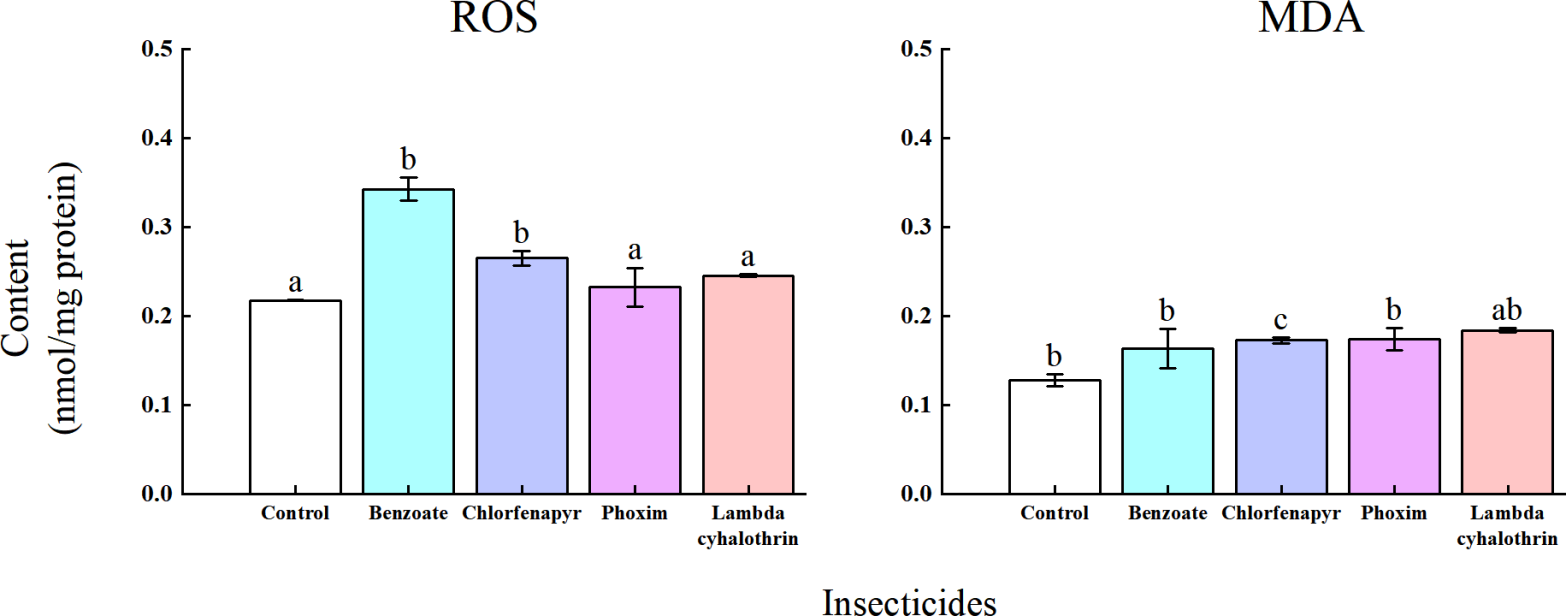
Sub-lethal concentration effects of the four insecticides on MDA and ROS content in adult *T. ostriniae.* Data are expressed as mean ± error and multiple comparisons among groups were performed using the Tukey test. Groups denoted by different letters represent significant differences (*P *< 0.05), whereas those denoted by the same letters indicate non – significant differences (*P *> 0.05). MDA, malondialdehyde; ROS, reactive oxygen species.

Following treatment with the four insecticides, the ROS level in the emamectin benzoate- and chlorfenapyr-treated groups was significantly higher than that in the control group (*P *< 0.05; Fig 5). The MDA level in the chlorfenapyr treatment group increased significantly, exhibiting a 1.39-fold increase compared with that in the control group (*P *< 0.05; Fig 5). The emamectin benzoate-treated group exhibited the highest ROS level (Fig 5). These findings suggest that chlorfenapyr induces the accumulation of ROS and MDA, leading to subsequent damage to *T. ostriniae* growth and development.

### Effects of four insecticides on the MRCC I and ATP levels in *T. ostriniae*

**Fig 6:**
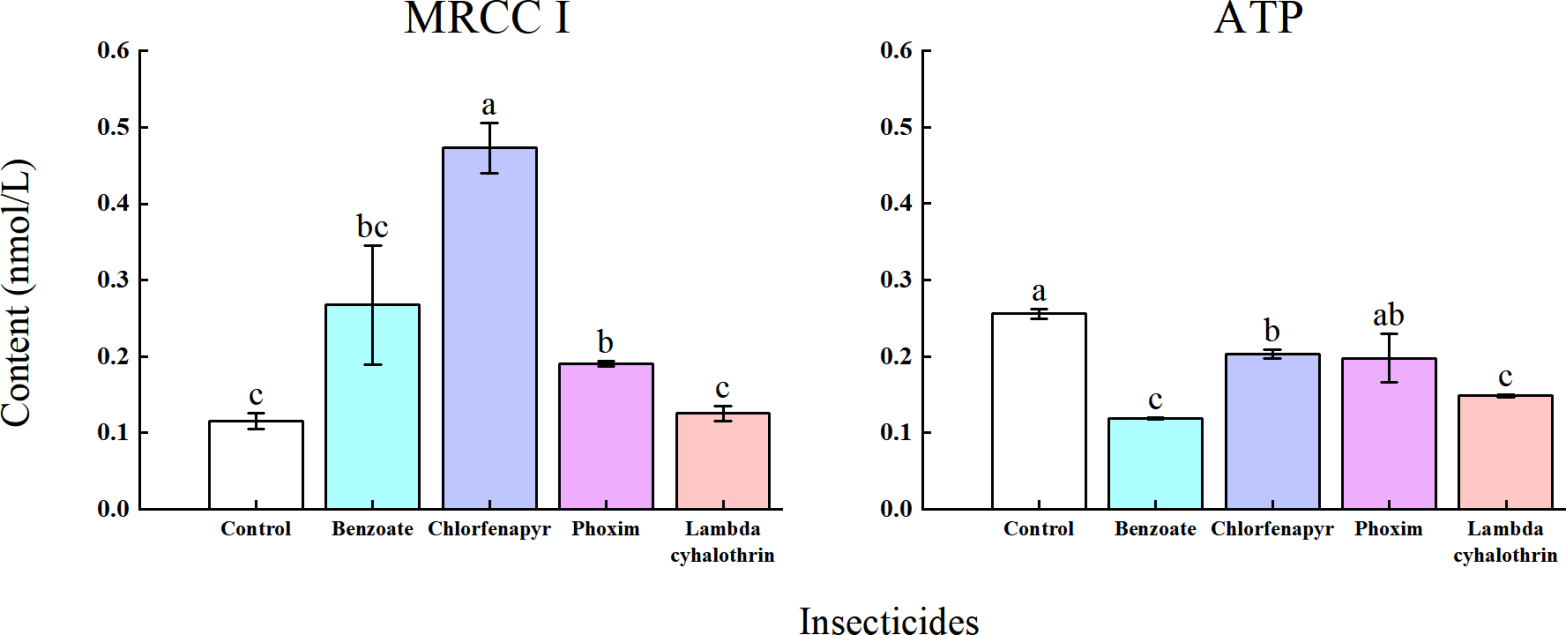
Sub-lethal concentration effects of four insecticides on MRCC I and ATP content in adult *T. ostriniae.* Data are expressed as mean ± error and multiple comparisons among groups were performed using the Tukey test. Groups denoted by different letters represent significant differences (*P *< 0.05), whereas those denoted by the same letters indicate non – significant differences (*P *> 0.05). MRCC I, mitochondrial respiratory chain complex I; ATP, adenosine triphosphate.

Treatment with the four insecticides resulted in a significant increase in MRCC I content in the chlorfenapyr and phoxim groups relative to those in the control group *(P *< 0.05; Fig 6). Specifically, the MRCC I levels were 4.08 and 1.64 times higher in the chlorfenapyr and phoxim groups, respectively, than in the control group. Additionally, the ATP levels in *T. ostriniae* were significantly reduced following treatment with lambda-cyhalothrin, chlorfenapyr, and emamectin benzoate relative to that in the control group *(P *< 0.05; Fig 6). The emamectin benzoate-treated group had the lowest ATP level (Fig 6).

## Discussion

Pesticides are widely used in agricultural ecosystems and can significantly affect the effectiveness of biological control agents. This is primarily due to the direct mortality of exposed arthropods and various sub-lethal physiological and behavioral effects [15]. Sub-lethal concentrations of insecticides can induce changes in beneficial insects, including alterations in weight, morphology, longevity, development duration, and fecundity, as well as shifts in population dynamics [20]. Our study assessed the acute lethal and sub-lethal effects of phoxim, lambda-cyhalothrin, emamectin benzoate, and chlorfenapyr and indicated that the four insecticides exhibited lethal toxicity in *T. ostriniae*. Emamectin benzoate is classified as a high-risk insecticide, whereas chlorfenapyr, phoxim, and lambda-cyhalothrin are considered to be extremely high-risk insecticides.

Our study demonstrated a significant reduction in the emergence rate following the application of the four insecticides at the larval and pupal stages, relative to the control group. Additionally, a significant reduction in the emergence rate was observed in the emamectin benzoate and chlorfenapyr groups at the egg, larval, pre-pupal, and pupal stages compared with the control group. These results are consistent with those of previous studies. Numerous studies have shown that different insecticides can reduce the emergence rates of egg parasitoids. For example, Costa *et al.* discovered that the application of various insecticides, including lambda-cyhalothrin + thiamethoxam, triflumuron, spinosad, fipronil, and thiamethoxam, led to a significant reduction in the emergence of *Trichogramma galloi* Zucchi, with the most pronounced effect observed when these were applied during the pupal stage [21]. Similarly, Souza *et al.* found that chlorpyrifos, beta-cypermethrin, spinosad, and chlorfenapyr diminished the emergence success of F1 *Trichogramma pretiosum* Riley [22]. Insects treated with etofenprox and alpha-cypermethrin/teflubenzuron during the egg-larval period and lambda-cyhalothrin/thiamethoxam during the pupal stage also experienced reduced emergence [22]. Furthermore, Moura *et al.* observed no adult emergence when insecticides were applied at the *T. pretiosum* pupal stage [23]. Organophosphates, such as chlorpyrifos-methyl, which are known for their high toxicity and ability to penetrate the insect cuticle, can easily contact the developed pupae beneath the eggshell [24]. Takada *et al.* (2001) and Turchen *et al.* (2016) found that insecticide residues on the host eggshell can inhibit adult emergence of *Trichogramma parasitoids* Westwood even when applied one day prior to emergence [25,26]. Therefore, selecting lesser toxic pesticides that are compatible with *Trichogramma* field releases is crucial for IPM programs that target lepidopteran crop pests.

Pesticides have been reported to negatively affect the reproductive performance of *Trichogramma* spp.; previous studies have indicated that insecticides, including pyrethroids, neonicotinoids, and organophosphates, can markedly diminish the fecundity of natural insect enemies, as evidenced by decreased oviposition and impaired discrimination of host eggs [27,28]. In the present study, the four insecticides markedly affected the fecundity of *T. ostriniae*. Specifically, lambda-cyhalothrin, emamectin benzoate, and chlorfenapyr exhibited a significant decrease in parasitism efficiency and maximum parasitism number of *C. cephalonica* eggs, whereas phoxim significantly enhanced both parameters.

Additionally, we found that the prey-handling time for *C. cephalonica* eggs by *T. ostriniae* was extended by lambda-cyhalothrin, emamectin benzoate, and chlorfenapyr; however, it was shortened by phoxim. This finding aligns with that of previous studies. For instance, Ray *et al.* (2023) reported that exposure of *T. chilonis* to imidacloprid resulted in a reduction in handling time and an increase in searching efficiency, per capita parasitization efficiency, and parasitization rate per handling time across multiple generations (F1–F5) when various densities of *C. cephalonica* eggs were used [29]. Similarly, Zhang *et al.* found that sub-lethal concentrations of azoxystrobin significantly reduced the predicted predation size, predation rate, and searching efficiency [30]. In this study, we observed that the searching efficiency of *T. ostriniae* on *C. cephalonica* eggs decreased with increasing egg density, eventually approaching control levels. This finding aligns with the effect of sub-lethal doses of imidacloprid on the predatory capacity of *Hippodamia variegata* Goeze [31]. These findings suggest intraspecific competition and mutual interference between *T. ostriniae*. Therefore, when applying *T. ostriniae* for pest control in the field, it is crucial to release it in a manner that considers species and host density to achieve optimal pest control efficacy while minimizing costs.

Although chemical control continues to be an effective pest management strategy, widespread and intensive use of insecticides has resulted in resistance to various major classes of insecticides in numerous field pest populations [32]. The emergence of insecticide resistance may be attributed to enhanced metabolic detoxification mediated by enzymes, including CYP450, GSTs, and CarEs [33,34]. CAT, SOD, and POD are essential protective enzymes in insects that are crucial for balancing free radicals and preventing free radical-induced toxicity [35]. Exposure to pollutants can lead to changes in the activity of these protective enzymes, which indicate the level of oxidative stress and serve as a marker of alterations in the physiological state of an organism [36]. Insecticides can either stimulate or suppress the insect protective enzyme system to various extents [37,38]. Previous studies have shown that increased CarE levels are associated with resistance to organophosphates and carbamates in *Schizaphis graminum* Rondani [39] and *Rhopalosiphum padi* Linnaeus [40]. Additionally, the enzymatic activities of CarE and GST in *Tetranychus urticae* Koch significantly increased 12 h after exposure to sub-lethal concentrations of abamectin (LC_10_ and LC_25_) [41]. Protective enzyme activity is a vital biomarker for the sub-lethal effects of insecticides. Previous research has found that exposure to an LC_25_ dose of emamectin benzoate, lambda-cyhalothrin, imidacloprid, or indoxacarb leads to a decline in these enzyme activities during the larval stage and subsequent development of *Chilo suppressalis* [42]. Consistent with previous research, our study found that, following treatment with emamectin benzoate, SOD and POD activities were elevated relative to those in the control group. Furthermore, the activities of CarEs, GSTs, and CYPs in *T. ostriniae* significantly increased following treatment with phoxim, chlorfenapyr, and emamectin benzoate compared with the control group.

Herbicides and insecticides also enhance ROS production in insects [43]. ROS are detrimental to biological systems because of their ability to oxidize lipids and damage proteins and DNA [44]. ROS-initiated lipid peroxidation results in the formation of MDA. Excessive MDA build-up can lead to structural alterations in intestinal cells, mitochondrial dysfunction, decreased bioenergetics, and oxidative stress [45]. MRCC I is a key site for ROS generation and is instrumental in driving ATP synthesis [46]. ATP is a vital high-energy phosphate molecule in living cells that supplies energy necessary for various cellular functions. Decreased ATP levels can result in reduced biological activity [47]. A previous study demonstrated that sub-lethal doses of tolfenpyrad can diminish the MRCC I level in *C. sinica* larvae, disrupting the balance between ROS production and elimination, thereby causing cellular damage [48]. Zhang *et al.* reported an increase in MDA and ROS content in *Chrysoperla sinica* Tjeder larvae after exposure to sub-lethal doses of tolfenpyrad, with continuous accumulation in the larvae [48]. In this study, we observed a significant increase in ROS levels in the emamectin benzoate- and chlorfenapyr-treated groups, whereas MDA levels were significantly increased in the chlorfenapyr-treated group. The MRCC I content of *T. ostriniae* increased considerably in the chlorfenapyr- and phoxim-treated groups. Additionally, the ATP level of *T. ostriniae* was significantly reduced after treatment with lambda-cyhalothrin, chlorfenapyr, and emamectin benzoate.

However, we only investigated the effects of these four insecticides on the biological traits of *T. ostriniae* under laboratory conditions. Further semi-field and field studies are required to fully understand their compatibility with the *Trichogramma* species.

## Conclusion

The four insecticides exhibited lethal toxicity to *T. ostriniae*. Emamectin benzoate was classified as a high-risk insecticide, whereas chlorfenapyr, phoxim, and lambda-cyhalothrin were considered as extremely high risk. To the best of our knowledge, this study is the first to reveal the effects of these four insecticides on reproduction, parasitism ability, detoxification enzymes, protective enzymes, and active substances in *T. ostriniae*. The integration of these insecticides into IPM strategies should be planned meticulously.

## Supporting information

S1 Data The minimal data set. (XLSX)
